# Evaluation of the benefits of plant growth-promoting rhizobacteria and mycorrhizal fungi on biochemical and morphophysiological traits of *Aloe barbadensis* Mill under water deficit stress

**DOI:** 10.1038/s41598-024-64878-9

**Published:** 2024-06-24

**Authors:** Rahil Khajeeyan, Amin Salehi, Mohsen Movahhedi Dehnavi, Mohammad Hamidian, Saeid Hazrati

**Affiliations:** 1 Department of Agronomy and Plant Breeding, Faculty of Agriculture, Yasouj University, Yasouj, Iran; 2 Department of Agronomy, Faculty of Agriculture, Azarbaijan Shahid Madani University, Tabriz, Iran

**Keywords:** Aloe, Antioxidant enzyme, Biofertilizers, Water stress, Nutrients, Plant sciences, Plant physiology, Plant signalling, Plant stress responses

## Abstract

*Aloe barbadensis* is a drought-tolerant perennial medicinal plant with both nutritional and cosmetic uses. Drought is one of the main abiotic stresses limiting plant growth and development. However, the use of drought-resistant plants combined with beneficial soil micro-organisms could improve the effectiveness of biological methods to mitigate drought damage. This research aims to evaluate the effects of *Funneliformis mosseae* (MF), plant growth-promoting rhizobacteria (PGPR) (including *Pseudomonas putida* and *Pantoea agglomerans*), and their co-inoculation on the macronutrient status, antioxidant enzyme activities, and other morphophysiological traits of *A. barbadensis* under four irrigation regimes [25%, 50%, 75% and 100% of water requirement (WR)]. Three harvests were conducted, revealing that inoculation enhanced the survival rate and shoot fresh weight (SFW) compared to the control plants. However, at 25% WR, the SFW was reduced by 43% more than the control. across all harvests, while the PGPR + MF treatment showed increases of more than 19%, 11%, and 17% compared to the control, MF, and PGPR treatments, respectively. The results also showed that *A. barbadensis* exhibited innate drought tolerance up to a 50% WR level by enhancing physiological defenses, such as antioxidant enzyme activity. Inoculation increased the macronutrient status of the plant at all levels of irrigation regimes especially under severe drought conditions. The highest levels of nitrogen (N) (16.24 mg g^−1^ DW) and phosphorus (P) (11.29 mg g^−1^ DW) were observed in the PGPR + MF treatment at 100% WR. The maximum relative water content under MF inoculation and 75% WR (98.24%) (98.24%) was reached. PGPR + MF treatment alleviated drought-induced osmotic stress, as indicated by reduced antioxidant enzyme activities and electrolyte leakage. However, *P. putida* and *P. agglomerans* strains alone or in combination with *F. mosseae* increased plant yield, macronutrient uptake and antioxidant enzyme activity. This study underscores the potential of these PGPR and MF strains as invaluable biological tools for the cultivation of *A. barbadensis* in regions with severe drought stress.

## Introduction

*Aloe vera* (*Aloe barbadensis*) Miller. is a succulent perennial drought-tolerant plant that belongs to the Xanthorrhoeaceae family, which includes more than 548 species^1^. The leaves of this plant, as the main part of the gel accumulation, are considered its commercial product. Due to the moisturizer properties and skin-beneficial natural compounds of its gel, there is an ever-increasing demand for skin care products and cosmetics for *A. barbadensis*^2^. In addition to the skin protection benefits, some other medicinal properties such as antioxidant, antimicrobial, and blood glucose and cholesterol regulation, and anti-inflammatories, have led to the expansion of the medical use of *A. barbadensis*^3^. On the other hand, besides the physio-chemical properties of *A. barbadensis* gel, the presence of some health-beneficial bioactive compounds, like acemannan, led to the introduction of *A. barbadensis* gel in the food industry and processing^4^. Therefore, this plant has commercial value in many respects, of its various medical, nutritional, and cosmetic uses in a variety of industries^5^.

The growth and yield of *A.* *barbadensis* depends on several factors such as climate, soil, and irrigation conditions, of which the amount of water available is the most important factor^6^. However, research have has shown that *A. barbadensis* plants have a reasonable potential for heat and drought tolerance^7,8^. The basis for this is related to the crassulacean acid metabolism (CAM) photosynthetic pathway and mechanism, which results in high water productivity and, consequently, better growth under stressful conditions^9^. In general, the negative effect of water deficit on the physiology of *A.* *barbadensis* leads to reduced yield and productivity due to the disruptions in the natural leaf growth rate^10^. For example, an increase in electrolyte leakage and a reduction in the relative water content of the plant leaves can be considered as one of these negative consequences during drought stress^11,12^. In addition, nutrient uptake, transport, and redistribution (especially P and N) are limited under drought stress due to reduced soil moisture, element availability, the release of these nutrients from soil colloids, and reduced root development^13^. These macronutrients are essential to plants. N is one of the most important elements in the pigment structure of plants; therefore, this nutrient has significant effects on photosynthesis and other critical physiological processes^12^. P is required for plant growth, N-fixation, energy metabolism, photosynthesis, and protection against environmental stresses. K plays an essential role in osmotic regulation, stomatal opening, and membrane integrity^14,15^.

Plants begin to close the stomata to control water loss by reducing transpiration under drought stress. Subsequently, the mesophyll tissue undergoes some reduction in CO_2_ concentration, and thereby, the dark reaction of photosynthesis is disturbed where the products of the light reaction, including ATP and NADPH, are not used. The lack of oxidation in the NADPH molecules causes decreasing the use of NADP to receive electrons. Consequently, oxygen molecules situated in the trajectory of the electron transfer function as recipients for electron substitution, thereby instigating the generation of superoxide radical (O^−^_2_), hydrogen peroxide (H_2_O_2_), and hydroxyl radical (˚OH)^16^. Enzymatic antioxidants, such as superoxide dismutase (SOD), catalase (CAT), and peroxidase (PRX), serve as protective measures against oxidative stress in plants. These mechanisms can scavenge reactive oxygen species, thereby reducing oxidative damage to the plant^17^. Naturally, the enzymatic activities of PRX and CAT are increased by increasing levels of stress, and plants with higher levels of CAT and PRX activities show improved drought tolerance^18^.

Biofertilizers are known as one of the bases of the sustainable agriculture perspective and as an important eco-friendly alternative to agrochemicals in the cultivation of medicinal plants^19^. Two types of biofertilizers, namely plant growth-promoting rhizobacteria (PGPR) and mycorrhizal fungi (MF), can promote stress tolerance in plants. Among various MF species, *Funneliformis mosseae* is considered to be one of the most suitable species for plants root mycorrhization^20,21^. In this light, Tawaraya et al. (2007)^22^ have reported that MF increase plant growth by improving the uptake of P and other nutrients; moreover, these fungi can produce growth hormones and boost rhizosphere biodiversity. Consequently, the damages of drought stress are mitigated in plants inoculated with mycorrhizae^23,24^. Previously, it has been reported that N and P uptake can be enhanced in *A. barbadensis* mycorrhizal plants^22^. In addition, several studies have shown that the improvement of enzymatic antioxidant defense by the application of PGPR and MF reduces abiotic stress damage in many plants^25–27^. On the other hand, there are some reports indicating that the antioxidant enzyme activity decreases or does not change significantly after MF and PGPR inoculation, due to the improvement of plant water conditions under stress^28–31^. Although this type of physiological response can be influenced by the types and species of beneficial rhizosphere microorganisms, plant species also play a crucial role. Nevertheless, there is limited information on the physiological responses of CAM plants (especially *A. barbadensis*) to PGPR under drought stress conditions.

**Figure 1:**
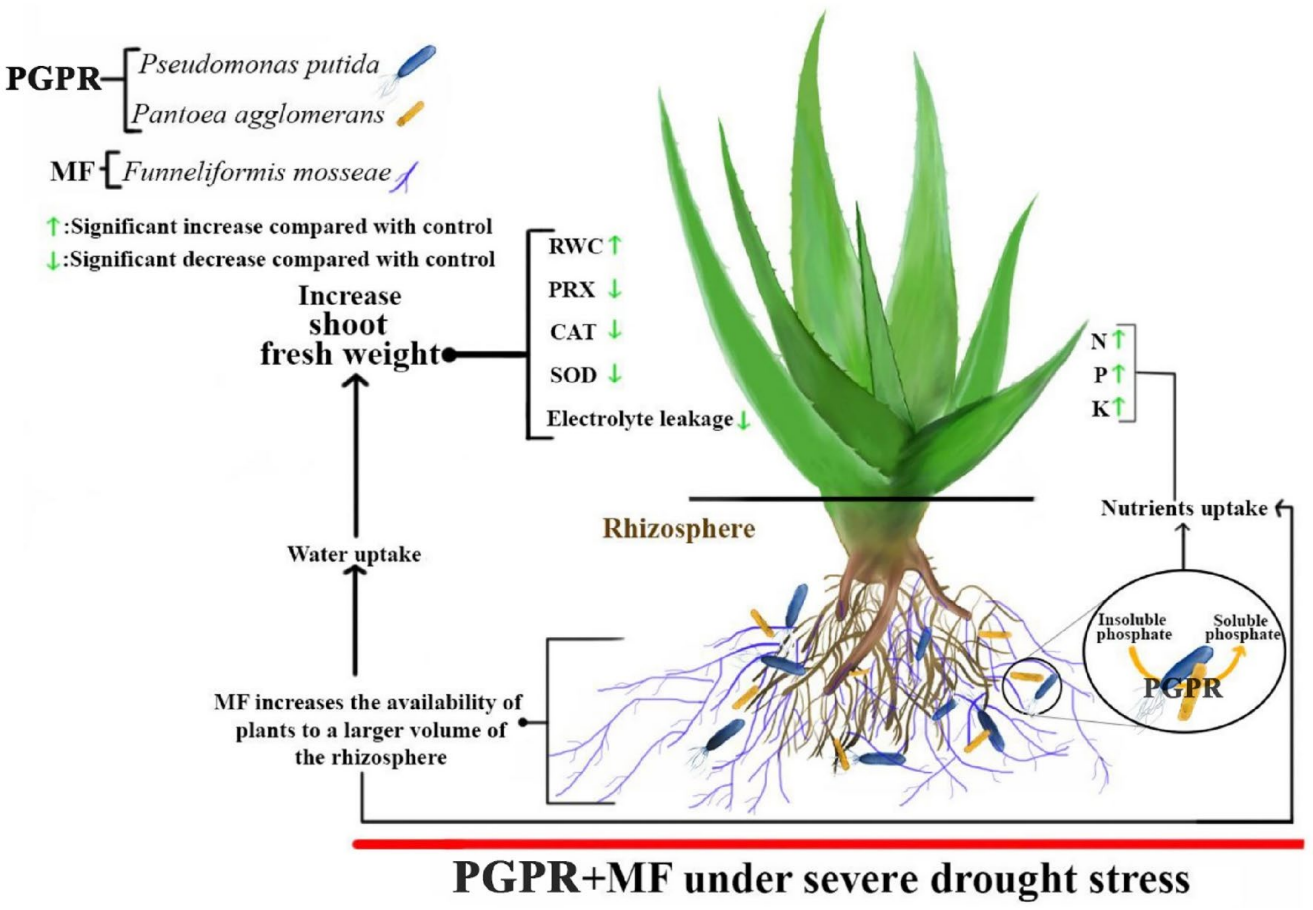
Graphical preview of physiological and growth variations of *Aloe barbadensis* under experimental factors. MF: Arbuscular Mycorrhiza, PGPR: plant growth-promoting hizobacteria, SOD: Superoxide dismutase, PRX: peroxidase and CAT: catalase.

Regarding to previous studies, it can be argued that biofertilizers can enhance plant growth and physiological responses, especially under stressed conditions. However, although previous studies have indicated the drought tolerance potential of *A. barbadensis*, the morphophysiological changes of this plant to plant-beneficial rhizospheric microorganisms under water stress may provide practical insights for *A.* *barbadensis* cultivation, especially in arid regions. Therefore, this study aimed to evaluate the potential of *F.* *mosseae* and two strains of phosphate solubilizing bacteria (PGPRs) (*Pseudomonas putida* and *Pantoea agglomerans*) as biofertilizers on the tolerance and yield of *A.* *barbadensis* under different water stress conditions in one of the semi-arid regions of Iran. To this end, enzymatic antioxidant defences (CAT, SOD and PRX), macronutrients (N, P and K) and other stress-related traits were investigated and briefly presented in Fig. 1.

## Results

### Nitrogen content

The two-way interaction between irrigation regimes and biofertilizers significantly influenced the N content in the three harvests (Table 1). Comparing the means of all harvests, indicated that the highest and lowest content of N were found in 100% of WR with PGPR + MF and 25% of WR without any biofertilizer, respectively. In addition, there was a significant difference between the biofertilizer treatments in all irrigation regimes. Co-inoculated with MF + PGPR was the best treatment for N uptake, especially under drought stress conditions. Compared with the control, this treatment increased the amount of leaf N content by 85% under 25% of WR in all three harvests (Table 2). In addition, leaf N content was slightly higher in the second harvest than in the other two harvests (Table 2).

**Table 1:** Analysis of the variance regarding the impact of biofertilizers and irrigation regimes on the leaf nutrient content and root colonization of *A. barbadensis* in three harvests.

S.O.V	df	N content			P content			K content			Root colonization
		H1	H2	H3	H1	H2	H3	H1	H2	H3	H3
Replication (R)	2	0.70*	0.51^ns^	0.98*	0.02^ns^	0.008^ns^	0.08^ns^	15.22*	12.86*	17.60*	47.27*
Irrigation regimes (I)	3	101.40**	111.90**	106.20**	3.44**	7.68**	4.51**	61.31**	56.25**	70.73**	747.52**
Error 1	6	0.81	0.79	0.99	0.07	0.09	0.11	2.69	3.36	3.09	17.93
Biofertilizers (B)	3	73.07**	78.82**	76.32**	17.51**	37.45**	19.23**	83.17**	72.91**	98.18**	3147.35**
R × B	6	1.11	1.17	0.98	0.54	1.22	0.74	3.13	2.46	3.83	53.77
I × B	9	1.62**	1.78**	1.68**	0.18*	0.43*	0.22*	7.91^ns^	7.94^ns^	9.10^ns^	31.81*
Error 2	18	0.18	0.21	0.22	0.06	0.15	0.08	3.79	3.26	4.34	9.95
C.V. %		4.19	4.30	4.56	4.14	4.50	4.57	7.83	7.36	7.75	8.44

ns,* and ** are no significant, significant at 5 and 1% probability levels, respectively. H1, H2 and H3: represents the first, second, third harvests, respectively.

**Table 2:** Mean comparison of the effect of biofertilizers in each level of irrigation regimes (interaction) on N and P contents in *A. barbadensis.*

Irrigation regimes (water requirement)	Biofertilizers	N content (mg g^−1^ LDW)			P content (mg g^−1^ LDW)		
		H1	H2	H3	H1	H2	H3
100%	Control	9.29d	9.75d	9.59d	5.02d	7.20d	5.32c
	MF	13.38b	13.90b	13.65b	7.05b	10.57b	7.80a
	PGPR	11.35c	11.80c	11.64c	5.72c	8.57c	6.06b
	PGPR + MF	15.62a	16.24a	15.93a	7.53a	11.29a	7.98a
75%	Control	8.96d	9.31d	9.13d	4.80d	7.20c	5.02c
	MF	13.32b	14.16b	13.58b	7.15b	10.73a	7.57a
	PGPR	11.13c	11.68c	11.26c	5.55c	8.32b	5.88b
	PGPR + MF	15.19a	15.96a	15.73a	7.59a	11.05a	8.04a
50%	Control	6.22d	6.62d	6.49d	4.24c	6.36c	4.71c
	MF	11.39b	11.81b	11.89b	6.98a	10.47a	7.39a
	PGPR	10.41c	11.01c	10.62c	5.37b	8.06b	5.69b
	PGPR + MF	13.26a	13.79a	13.52a	7.38a	10.74a	7.82a
25%	Control	4.27d	4.44d	4.37d	4.05c	6.08c	4.29c
	MF	6.48b	6.77b	6.66b	5.69a	8.54a	6.01a
	PGPR	5.67c	5.90c	5.79c	4.84b	7.27b	5.13b
	PGPR + MF	8.03a	8.39a	8.21a	6.08a	8.82a	6.25a

Values in the same column sharing different letters are significantly different (p < 0.05). (Control: without biofertilizers; MF: mycorrhizal fungi; PGPR: plant growth-promoting rhizobacteria; H1, H2 and H3: represents the first, second, third harvests, respectively).

### Phosphorous content

The results showed that the interaction between irrigation regimes and biofertilizers significantly affected the P content in the leaves (Table 1). The mean comparisons showed that the highest amount of leaf P content was observed in 75% of WR with PGPR + MF treatment. Furthermore, a significant difference was observed between the other biofertilizers at this irrigation level. The plants were grown under 25% of WR without any biofertilizer had the lowest P content among the treatments. Similar to the changes in N content in the second harvest, the highest amount of P was evaluated in this harvest (Table 2). In general, MF had a significantly affected on P content than PGPR in this study. However, the highest P content was obtained in the combined PGPR + MF treatment. In fact, in the PGPR + MF treatment under 25% of WR, the leaf P content was about 50.1, 45.0, and 45.6% higher than in the control plants in the first, second, and third harvests, respectively.

### Potassium content

Irrigation regimes and biofertilizers significantly affected the K content in leaves, while their interaction was insignificant (Table 1). The means comparison showed a higher level of K when the plants were used severe water stress. In fact, the highest amount of K content was found at 25% of WR irrigation level. Additionally, there was a significant difference between 25% of WR and the other irrigation regimes in terms of K content (Table 4). The application of the MF treatment resulted in a significant positive difference in K content compared to the PGPR and the control (Table 4). However, the highest K content was obtained in the PGPR + MF treatment, in which the K content was significantly higher than other biofertilizer treatments. In addition, the amount of this element in the three harvests was about 25–26% higher in the plants inoculated with MF and PGPR than in the control (Table 4).

### Enzymes (catalase, peroxidase, and superoxide dismutase) activity

In all three harvests, it can be observed that the enzyme activities were significantly influenced by the irrigation regimes and biofertilizers, while their interaction had no significant effect on the enzyme activities (Table 3). Severe water stress led to the emergence of higher amounts of CAT (Table 4), PRX, and SOD activities (Table 5). In fact, the activity of CAT, SOD, and POD under severe stress conditions (25% WR) in all harvests was at least 74.8, 46, and 57% higher than 100% WR in all plants (Tables 4 and 5). The application of biofertilizers resulted in reduced enzyme activity. PGPR + MF treatment significantly reduced the enzyme activities significantly compared to other biofertilizers and the same trends were observed in all three harvests (Tables 4 and 5).

**Table 3:** Analysis of the variance regarding the impact of biofertilizers and irrigation regimes on the enzyme activity of *A. barbadensis* leaf in three harvests.

S.O.V	df	CAT			PRX			SOD		
		H1	H2	H3	H1	H2	H3	H1	H2	H3
Replication (R)	2	0.67**	0.62**	0.66**	5.19^ns^	4.97^ns^	5.00^ns^	15.14*	14.01*	14.40*
Irrigation regimes (I)	3	10.80**	9.94**	10.53**	241.8**	224.5**	232.1**	314.0**	289.2**	301.5**
Error 1	6	0.05	0.04	0.05	4.45	4.15	4.28	5.50	5.07	5.24
Biofertilizers (B)	3	13.05**	12.03**	12.61**	212.2**	194.8**	203.8**	255.4**	235.4**	245.1**
R × B	6	0.13	0.12	0.14	2.70	2.54	2.59	5.73	5.28	5.57
I × B	9	0.15^ns^	0.14^ns^	0.14^ns^	5.50^ns^	5.04^ns^	5.27^ns^	3.25^ns^	3.01^ns^	3.09^ns^
Error 2	18	0.10	0.09	0.10	3.34	3.13	3.21	3.22	2.96	3.11
C.V		9.12	9.10	9.05	8.72	8.78	8.71	5.79	5.79	5.80

ns,* and ** are no significant, significant at 5 and 1% probability levels, respectively. H1, H2 and H3: represent the first, second, third harvests, respectively. CAT: catalase enzyme; PRX: peroxidase enzyme; SOD: superoxide dismutase enzyme.

**Table 4:** Means comparison of the main effect of irrigation regimes and biofertilizers on K content and CAT activity in *A. barbadensis.*

	K content (mg g^-1^ LDW)			CAT activity (mM.g^-1^.min^-1^)		
	H1	H2	H3	H1	H2	H3
Irrigation regimes (water requirement)						
100%	22.78c	22.52c	24.62c	2.82c	2.70c	2.76c
75%	23.77bc	23.56bc	25.73bc	3.03c	2.91c	2.97c
50%	24.89b	24.48b	26.91b	3.55b	3.40b	3.48b
25%	27.99a	27.55a	30.23a	4.93a	4.73a	4.85a
Biofertilizers						
Control	22.62c	22.31c	24.43c	4.78a	5.59a	4.69a
MF	25.11b	24.70b	27.13b	3.86b	3.70b	3.79b
PGPR	23.24c	23.19bc	25.12c	3.42c	3.28c	3.36c
PGPR + MF	28.47a	27.91a	30.80a	2.27d	2.18d	2.22d

Values in the same column sharing different letters are significantly different (p < 0.05). (Control: without biofertilizers; MF: mycorrhizal fungi; PGPR: plant growth-promoting rhizobacteria; H1, H2 and H3: represents the first, second, third harvests, respectively). Ldw: leaf dry weight; CAT: catalase enzyme.

**Table 5:** Mean comparison of the main effect of irrigation regimes and biofertilizers on PRX and SOD activities in *A. barbadensis.*

	PRX activity (µM.g^-1^. min^-1^)			SOD activity (IU.mg^-1^)		
	H1	H2	H3	H1	H2	H3
Irrigation regimes (water requirement)						
100%	17.29c	16.61c	16.95c	25.84d	24.81d	25.33d
75%	18.11c	17.38c	17.75c	28.63c	27.49c	28.07c
50%	21.33b	20.48b	20.91b	31.72b	30.45b	31.09b
25%	27.18a	26.14a	26.64a	37.77a	36.25a	37.02a
Biofertilizers						
Control	26.87a	27.79a	26.33a	36.39a	34.93a	35.66a
MF	20.52b	19.71b	20.11b	32.61b	31.30b	31.97b
PGPR	19.58b	18.84b	19.19b	29.43c	28.25c	28.85c
PGPR + MF	16.95c	16.28c	16.61c	25.54d	24.52d	25.04d

Values in the same column sharing different letters are significantly different (p < 0.05). (Control: without biofertilizers; MF: mycorrhizal fungi; PGPR: plant growth-promoting rhizobacteria; H1, H2 and H3: represents the first, second, third harvests, respectively). PRX: peroxidase enzyme; SOD: superoxide dismutase enzyme.

### Relative water content (RWC)

RWC in *A. barbadensis* leaves was significantly influenced by the interaction of irrigation regimes and biofertilizers in all the harvests (Table 6). In the first and third harvests, the RWC in the control plants was significantly lower than in PGPR, MF and their combination. Furthermore, this trend was exacerbated by increasing stress levels. For example, based on the results obtained from all harvests under severe water stress levels (25% WR), RWC increased by more than 22% in PGPR + MF treatments rather than the control; however, this amount was 7.9% at 50% WR leveld (Table 8).

**Table 6:** Analysis of the variance regarding the impact of biofertilizers and irrigation regimes on the RWC, electrolyte leakage, and shoot FW of *A. Barbadensis* in three harvests.

S.O.V	df	RWC			Electrolyte leakage			Shoot FW		
		H1	H2	H3	H1	H2	H3	H1	H2	H3
Replication I	2	9.22^ns^	11.80^ns^	9.40^ns^	19.0**	14.5^ns^	15.7*	1455467^ns^	1761115^ns^	2130949^ns^
Irrigation regimes (I)	3	79.94*	76.37*	81.60*	291.5**	269.2**	261.1**	64,407,818**	77,933,460**	94,299,486**
Error 1	6	11.58	10.27	11.81	6.0	9.5	4.5	4,121,687	4,987,242	6,034,562
Biofertilizers (B)	3	238.46**	234.45**	243.26**	624.0**	547.7**	570.5**	7,203,626*	8,716,388*	10,546,829*
R × B	6	16.64	15.93	16.98	32.3	39.5	29.7	2727536^ns^	3300319^ns^	3993386^ns^
I × B	9	24.11*	26.17*	24.59*	4.0^ns^	3.9^ns^	4.6^ns^	1455467^ns^	1761115^ns^	2130949^ns^
Error 2	18	7.13	7.65	7.26	3.1	4.6	2.9	64,407,818	1,997,976	2,417,550
C.V.%		2.86	2.91	2.86	3.76	4.84	3.69	15.54	13.72	13.93

ns,* and ** are no significant, significant at 5 and 1% probability levels, respectively. H1, H2 and H3: represents the first, second, third harvests, respectively.

#### Leaf electrolyte leakage

The results showed that the irrigation regimes had a significantly affected on the percentage of leakage (Table 6). The plants that received less water had a higher percentage of leakage. Based on the mean comparisons, 25% of the WR showed a higher amount of leakage, which was about 25–27% higher than 100% of the WR treatments in all three harvests (Table 7). This characteristic was also statistically influenced by the different biofertilizer treatments, as well (Table 7). In the control plants, the leaf leakage percentage increased was significantly higher than in the biofertilizer treatments. During the second harvest, the existing better climatic situation caused lower amounts of leakage in the leaves compared to the other harvests (Table 7).

**Table 7:** Mean comparison of the main effect of irrigation regimes and biofertilizers on electrolyte leakage and shoot fresh weight in *A. barbadensis.*

	Electrolyte leakage (%)			Shoot FW (kg. plant^-1^)		
	H1	H2	H3	H1	H2	H3
Irrigation regimes (water requirement)						
100%	42.00c	40.08c	41.75c	10.562a	11.618a	12.780a
75%	43.83c	42.00c	43.41c	11.065a	12.171a	13.389a
50%	48.41b	45.75b	47.66b	9.847a	10.831a	11.915a
25%	53.00a	50.83a	52.16a	5.967b	6.564b	7.220b
Biofertilizers						
Control	56.25a	53.41a	55.25a	8.737b	9.610b	10.571b
MF	45.41b	43.41b	45.00b	9.353b	10.289b	11.317b
PGPR	46.83b	44.83b	46.25b	8.896b	9.786b	10.765b
PGPR + MF	38.75c	37.00c	38.50c	10.454a	11.500a	12.650a

Values in the same column sharing different letters are significantly different (p < 0.05). (Control: without biofertilizers; MF: mycorrhizal fungi; PGPR: plant growth-promoting rhizobacteria; H1, H2 and H3: represents the first, second, third harvests, respectively).

### Shoot fresh weight (SFW)

The main effect of biofertilizer application and irrigation regimes on the SFW of *A. barbadensis* was significant (Table 6). Under water stress conditions, normal plant growth was disturbed; however, no significant difference was observed between 50%, 75% and 100% WR on SFW. This indicates an appropriate tolerance of *A. barbadensis* to drought. The most significant damage was observed in 25% WR in all three harvests compared to 100% WR. In fact, up to a 43.5% reduction in SFW was obtained in 25% WR compared to 100% WR. In general, the application of both types of MF and PGPR treatments separately had an insignificant effect on SFW compared to the control plants. However, the highest SFW was related to the combined PGPR + MF treatment, which was significantly higher than the other treatments (Table 7).

### Root colonization

**Figure 2:**
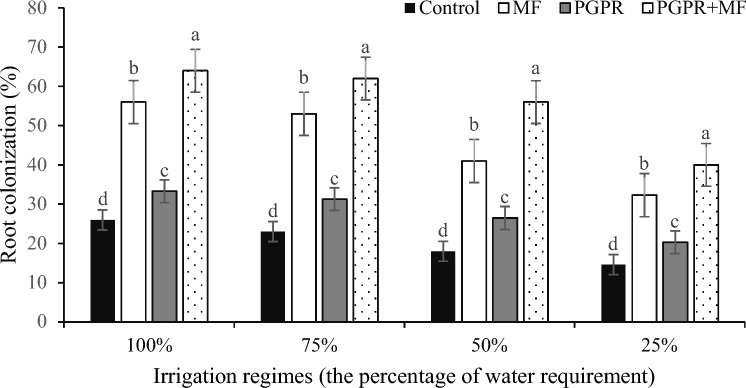
Mean comparison of different levels of irrigation regimes and biofertilizers at the end of the experiment (third harvest) for the percentage of root colonization; Control: no bio fertilizer, MF: mycorrhizal fungi, and plant growth-promoting rhizobacteria (PGPR): phosphate solubilizing bacteria; the same letters on each column have no significant difference (p < 0.05).

At the end of the experiment, root colonization percentages of *A. barbadensis* varied among the different irrigation regimes and biofertilizer treatments during the third harvest (Table 2). In particular, there was a significant increase in root colonization when the PGPR + MF treatment was applied under each irrigation regime compared to the other biofertilizer treatments (Fig. 2). While the well-watered treatments showed higher colonization rates, there was no significant difference between 100 and 75% WR (Fig. 2). The use of PGPR + MF resulted in the highest colonization percentages.

## Discussion

The results showed that water stress leads to reduced nutrient content in plants, which can be attributed to the lower nutrient uptake and transport in plants under water deficit. In general, the nutrient content of *A. barbadensis* can be influenced by different factors such as growth situation, soil, climate, geographical region, and plant age. One of these factors is water availability^32,33^. It has been observed that higher water availability leads to higher nutrient accumulation in *A. barbadensis* plants. Gonzalez-Dugo et al. (2010)^34^ reported that drought stress reduces plant N uptake even in soils with rich sources of N. Therefore, the current results regarding decreasing N content in plant shoots under water deficit are in line with other research^35,36^. Similar to the results of this study, it has been mentioned that biofertilizer sources, especially *F. mosseae*, have a significant effect on N and P uptake in *A. barbadensis*^37^. On the other hand, elevating P uptake in mycorrhizal plants may have a positive effect on other nutrient contents such as N under water deficit conditions^35^. Another study confirmed, the positive correlation between N and P has been confirmed^38^. The current results indicate that improving P levels by PGPRs can increase N accumulation (Table 2). Similarly, an increase in P and N uptake as a result of the use of PGPRs has been verified^39^. In addition, the results of another study on *A. barbadensis* indicated that the use of PGPR increased the availability of P in the soil and led to its accumulation in the leaves. The mentioned study was carried out on four different PGPRs, among which *pseudomonas* brought about the highest amount of P content in the plants^40^. In the present research, the *Pseudomonas* genus was one of the PGPRs. Likewise, Gupta et al.^40^ reported that PGPRs could elevate P availability for plants by means of adjusting soil pH and solubilizing mineral P through organic acid production (e.g., gluconic, ketogluconic, and oxalic acids) and phosphatase enzymes. Other research findings also support this claim that PGPR inoculation increases the P content^41^. The first solubilizing mechanisms of P in plants and microorganisms include H + exclusion, organic acid production, and acid phosphatase biosynthesis. Additionally, organic acids can increase P availability by inhibiting the reaction of P with other soil ions such as Ca^39^. An experiment on coneflower as a medicinal plant showed a higher level of K content in response to water stress, which may be due to the involvement of K in osmotic regulation, stomatal opening, and membrane permanence under drought stress^36^. In the current research, the higher K content under severe water stress supports the finding that the K content may act as an essential tolerance factor against stressful situations for *A. barbadensis* (Table 4). Moreover, the K content in *A. barbadensis* leaves was higher than the other two macro elements (N and P) 35. The same result was observed in the present study (Table 2). It has been reported that biofertilizers and MF can increase K content in *A. barbadensis* leaves by expanding the access of the plants to higher soil volumes.

Ramirez et al.^42^ confirmed that some changes, such as an increase SOD, occur in *A. barbadensis* plants, particularly in the apexes of leaves under water stress, which is similar to the current estimations. SOD is one of the enzymatic antioxidants that alter superoxide ions to hydrogen peroxide (H_2_O_2_)^43^. However, this product is also another oxygen radical form, and CAT and PRX play an important role in scavenging H_2_O_2_^44^. PRX exists in peroxisomes despite the presence of other enzymes in different parts of plants^43^. In this regard, Mohammadi et al. (2019)^45^ reported that water deficit stress would increase PRX in most plants, especially in arid and semiarid regions. Naturally, CAT activity would increase under drought stress. In addition, it has been reported that the activity of CAT enzyme was enhanced in mycorrhizal plants under water deficit^46^. On the other hand, Aguacil et al.^47^ posited that some species of MF may increase enzymatic activities such as CAT; on the contrary, other species may lead to a decrease in the production of this enzymatic activity. In addition, it has been shown that PGPR inoculation decreases antioxidant enzyme activity due to the promotion of plant growth and water status^44^. It has also been reported, in line with the findings of this study, that the activity of PRX^48^ and SOD^49,50^ activity can be reduced in plants inoculated with PGPR as a result of the mitigation and modulation of the environmental effects on plants by PGPR.

Leaf RWC was monitored to evaluate the water status of *A. barbadensis* according to the treatments applied. As the level of drought stress increased, RWC decreased in all treatments, especially in 25% WR. The combined treatment of PGPR + MF reduced the adverse effect of stress, resulting in a significantly increase the RWC at all stress levels, which was significant at severe stress (25% of WR) (Table 8). Under drought stress conditions, changes in osmotic potential in the soil and plant tissues as a result of water reduction have a negative effect on water uptake and transfer in plants. Therefore, traits related to plant water status, such as RWC, are reduced under drought stress^51^. Subramanian et al. (2006)^52^ reported that mycorrhizal plants prevent reduced RWC under drought stress. The beneficial effect of MF on RWC has been reported for other plants^11,53^. Plant root systems are often expanded and developed by mycorrhizal inoculation. Consequently, plants can have a larger absorptive area in the soil to seek and access water. In addition, MF contributes directly to water uptake by plants, especially under water-limited conditions, through its external hyphae. Similar to the results obtained (Table 8), the positive effect of PGPR in maintaining RWC under stress has been reported^54^. Electrolyte leakage demonstrates cell membrane damage under stressful situations. Water deficit stress can lead to a higher cell leakage level. The accumulation of toxic levels of active oxygen radicals due to drought stress damages the cell membrane lipids, proteins, and nucleic acid and, resulting in increased electrolyte leakage^55^.

**Table 8:** Means comparison of the effect of biofertilizers in each level of irrigation regimes (interaction) on RWC in *A. barbadensis.*

Irrigation regimes (water requirement)	Biofertilizers	RWC (%)		
		H1	H2	H3
100%	Control	90.41b	92.21a	91.31b
	MF	95.64a	97.55a	96.59a
	PGPR	95.22a	97.12a	96.17a
	PGPR + MF	96.89a	98.03a	97.86a
75%	Control	90.68b	92.49a	91.58b
	MF	96.32a	98.24a	97.28a
	PGPR	95.32a	97.22a	96.27a
	PGPR + MF	97.11a	97.98a	98.08a
50%	Control	88.71b	90.48b	89.59b
	MF	95.37a	97.28a	96.32a
	PGPR	95.84a	97.75a	96.79a
	PGPR + MF	96.48a	97.70a	97.44a
25%	Control	76.46b	77.99b	77.22b
	MF	93.52a	95.38a	94.44a
	PGPR	93.75a	95.62a	94.68a
	PGPR + MF	93.77a	95.64a	94.70a

Values in the same column sharing different letters are significantly different (p < 0.05). (Control: without biofertilizers; MF: mycorrhizal fungi; PGPR: plant growth-promoting rhizobacteria; H1, H2 and H3: represents the first, second, third harvests, respectively). RWC: relative water content.

It is very important to determine the stability and tolerance of growth responses under stressful conditions because it shows the capacity of a plant species to produce under limited water resource. On the other hand, the fresh shoot weight of *A. barbadensis* can be considered as main trait that reveals the productivity of this plant under different conditions. Similarly, the improvement of growth and productivity of medicinal plants by the application of PGPR and MF under drought stress has been reported previously^56,57^. In addition, many studies have found that combining PGPR and MF can be more effective than applying of these beneficial microorganisms individually under drought stress due to reduced oxidative stress, improving osmoregulation, nutrient hemostasis and increased water productivity^58,59^. This phenomenon lies in not only in the individual benefit of each microorganism to plants but also in the positive effects and cooperation of both on a healthier rhizosphere microbiome to modulate soil bacterial population and colonization for the benefit of plants^60^. On the other hand, Silva et al. (2010)^7^ demonstrated high water productivity in CAM plants compared to C3 and C4 species. Similarly, they claimed that biomass was significantly reduced under severe water stress and tended to increase the value of water productivity. In another study, lower water availability reduced the biomass of *A. barbadensis*^7^. However, the results of the present study showed that the application of soil microorganisms as biofertilizers effectively improved plant growth and increased plant biomass under lower water availability.

The MF colonization rate reflects the degree of infection and the affinity between the AM and the host plant. In the present study, the AM colonization rate was high under all the water stresses, indicating a relatively high affinity between the selected AM and *A. barbadensis*. Environmental and root conditions play a crucial role in mycorrhizal colonization. Previous studies by Subramanian et al.^52^ and Sheng et al.^53^ have shown that severe drought stress can lead to lower mycorrhizal colonization rates. In addition, the combined interaction between MF and rhizobacteria has been shown to enhance plant growth^61^. Our study also showed an increase in growth parameters under PGPB + MF at each irrigation level, highlighting this beneficial effect. Previous studies have suggested that mycorrhizal colonization can be increased by approximately 10% by the application of PGPR^61,62^. In our study, PGPRB + MF under different irrigation regimes resulted in the highest colonization percentage during the third harvest (Fig. 2). Therefore, it seems that under severe water stress accompanied by warmer and/or drier conditions (observed during the third harvests with average temperatures of 30.82 °C and 28.7 °C, respectively, and 0 mm rainfall for both periods), the combined influence of biofertilizers and water availability becomes more pronounced.

## Materials and methods

### Experimental design and treatments

This study was conducted between 2016 and 2018 in Iran (Bushehr Province) using a split-plot experiment based on randomized complete block design with three replications (50° 12′ E and 29° 16′ N; 8.40 m above sea level). The chemical and physical properties of soil in the depth of 0–30 cm were measured. The studied soil textures included sandy loam, organic carbon, N, P, and K with contents of 0.32%, 0.03%, 6.8 and 170 mg/kg, respectively. The mean values of annual temperature and long-term mean precipitation in the region were equal to 26.38 ℃ and 287 mm, respectively. The four levels of irrigation regimes (i.e., 25, 50, 75 and 100% of water requirement (WR)) were considered as the main factors, whereas the four levels of biofertilizers, namely arbuscular MF (*Funneliformis* *mosseae*) commercially available, plant growth-promoting rhizobacteria (PGPR) containing *Pseudomonas putida* (strain P13 with accession No. EU545414) and *Pantoea agglomerans* (strain P5 with Bio Project code: PRJNA386632), PGPR + MF, and control (without any biofertilizers), were regarded as sub-factors.

The plants were grown in a research field at the Faculty of Agriculture, Persian Gulf University, Bushehr, Iran, in 2015 and 2016. The 18–20 cm offsets (small plants growing from the sides of the mother plant) were planted in plastic pots and placed in a greenhouse for two months, irrigated equally. After this time, offsets were transplanted into the experimental field in October 2016. Since the plantlets had been established well, irrigation treatments were commenced and biofertilizer inoculation was performed during the planting process. There were five 3-m-long rows on each plot, with the plants spaced 50 cm apart within each row. The distances between the main plots, subplots, and blocks were 2.4, 1.2, and 2 m, respectively. Moreover, three leaf harvests were carried out in June 2017, December 2017, and June 2018.

Before planting, roots were washed completely. PGPR inoculation was also conducted by soaking the plantlet roots in a solution containing the recommended manufactured (*Zist Fanavar Sabz Company, Iran*) amount (100 g ha^−1^) of PGPR biofertilizers for 24 h. The concentration was adjusted to 10^8^ colony-forming units (CFU) mL^−1^. Mycorrhizal inoculum, obtained from the clinic production of organic plant protection of Asadabad in Hamadan with registration number 27.1554, Iran, was presented as an inoculant of MF (*F. mosseae*). Fungus inoculation was performed in rows near the plantlet roots, uniformly mixed into the soil before planting offsets with the recommended amount of manufactured biofertilizer (80 kg ha^−1^). Offsets were then carefully placed on the fungi inoculum, and soil was lightly poured around them (containing spore numbers of 120 g^−1^ substrate) at the planting time in October 2016. Non-inoculation with AM fungus also included supplying 150 g of autoclaved mycorrhizal inoculum to maintain consistent microbes except for the AM fungus. After the plantlets were established well (around six months after planting), irrigation treatments were applied. Irrigation was performed using the drip method with seven-day intervals depending on weather conditions to maintain favorable water conditions in the soil. Irrigation was based on daily evapotranspiration according to evapotranspiration requirements, and the plant water requirement measurements constituted the irrigation criteria based on the Penman–Monteith Equation^62^. For this purpose, the reference evapotranspiration of the plant (ETo (mm.day^−1^) was obtained, and the daily water requirements of the plant were determined through Eqs. 1, 2, and 3. In addition, water volume meter was used to determine the water volume. Finally, the amounts of water consumed for each treatment were respectively obtained to be 19,950, 14,964, 9975, and 4989 cubic meters per hectare from the beginning of the planting to the last harvest (2016–2018) in order to supply 100, 75, 50 and 25% of ETc for plant WR. Of the total annual rainfall in 2017 and 2018 (243.08 and 264.42 mm), approximately 193.31 and 187.09 mm of rain had fallen in January, February, and March, respectively. During these months, rainfall information was collected daily from the meteorological organization of Bushehr province. In the irrigation treatments, the amount of rainfall and evapotranspiration were considered and each treatment was calculated by.

Equations:

1 $$\text{ETc }=\text{ KC}\times \text{ETo}$$

2 $$\text{D }= \sum_{i=1}^{n}(ETci)$$

3 $$\text{V }=\text{ D }\times \text{ A}$$

ETc: Plant evapotranspiration (mm.day^−1^), KC: Crop coefficient; KC for *A. barbadensis* is 0.35^63^, D: Irrigation depth (m), Ʃ^n^_i=1_(ETci): Sum of the plant water requirements based on irrigation frequency, V: Irrigation volume (m^3^), A: Plot area (m^2^).

Different irrigation treatments were implemented by varying the emitter output per m^2^ in the drip line, ranging from 2 L h^−1^ to 16 L h^−1^. The irrigation pump ran based on the application rate of the 100% ETc treatment plots. Accordingly, the 25%, 50%, 75%, and 100% ETc plots received 2 L h^−1^, 4 L h^−1^, 8 L h^−1^, and 16 L h^−1^ per m^2^, respectively.

Three plants were completely harvested from the ground to measure the SFW in each harvest, and the total SFW was calculated. Subsequently, the first, second and third harvests were executed in June 2017, December 2017 and June 2018, respectively. One of the mature leaves from the three specified plants in each replication was cut and transferred to freezer -40 in liquid N to evaluate the physiological traits. This approach was repeated in all harvests.

### Nutrient measurements

The extract from the dried leaf for measuring the nutrients was obtained based on the digesting method in flasks with H_2_SO_4_-salicylic acid-H_2_O^64^. An alkaline medium was used to determine the N content by combining salicylate with ammonia and hypochlorite, resulting in a green–blue color indophenol. Eventually, the absorbance of samples was measured at 660 nm^64^. the extract P content was measured by changing the color in reacting molybdate-vanadate by spectrophotometer (Lambda EZ210, U.S.A.) at a wavelength of 420 nm. Similarly, K phosphate was used to prepare the standard P^65^. The content of K was read using the flame diffusion method utilizing a flame photometer in mg g^−1^ of the extract. For this reason, the samples were dried and placed in a 500 °C oven and converted into ash. Following the addition of HCl and the heating and addition of distilled water, the content of K was read using a flame photometer^66^. It was determined based on K standards (KCl). All nutrient contents were expressed as mg/g leaf dry weight.

### Enzyme activities

We homogenized the leaf sample at a low temperature in an extraction buffer, and after centrifugation, we measured enzyme activity in supernatant^67^. Catalase (CAT) activity was measured using the Cakmak and Horst (1991) method^68^. The reaction mixture comprised H_2_O_2_ (10 mM), sodium phosphate buffer (pH = 6.8), and 100 µL of the enzymatic extract. CAT activity was determined by a decrease in absorbance at 240 nm, and the result was reported as mmol g^−1^ min^−1^. Peroxidase enzyme (PRX) activity was determined according to the Zhang and Kirkham (1996) method^69^. In this process, the reaction mixture contained potassium phosphate buffer (60 mM with pH = 6.1), guaiacol (28 mM), H_2_O_2_ (5 mM), and 100 µL of the extracted solution. An increase in the absorbance was reported at 470 nm (by spectrophotometer (UV-2550, Shimadzu, Japan) where the amount of µmol g^−1^ min^−1^ was expressed. Superoxide dismutase (SOD) activity was assessed using the method by Beauchamp and Fridovich (1971)^70^. Enzyme activity measuring based on the inhibition of the photochemical reduction of nitroblue tetrazolium (NBT). In the reaction mix were 50 mM of phosphate buffer (pH 7.8, containing 0.1 mM of EDTA), 13 mM of methionine, 75 µM of NBT, and 4 µM of riboflavin, and 50 µl of enzyme extract. Enzyme activity was determined at 560 nm by comparing the reaction mix with the control solution.

### Relative water content (RWC)

The value was measured using Eq. 4 was expressed as the %^71^.

4 $$\text{RWC }=\text{ FW}-\text{ DW}/\text{TW}-\text{DW}$$

where FW is the leaf fresh weight (weighting the leaves immediately after harvesting), DW is the leaf dry weight, and TW is the leaf turgor weight (weighting after leaf floating in distilled water for 4–6 h).

### Electrolyte leakage

The electrolyte leakage was measured using Eq. 5 and the results were reported as the percentage^72^.

5 $$\text{Electrolyte leakage }= (\text{EC}1/\text{EC}2) \times 100$$

where EC_1_ is the electrical conductivity after the placement of the fresh tissue in 30 ml of distilled water in a dark place for 24 h; EC_2_ is the electrical conductivity after the placement of the fresh tissue in the boiling bath for 45 min and cooling it at normal room temperature for 24 h.

### Colonization determinations

To assess the efficiency of mycorrhizal inoculation, the percentage of root length colonization was determined. Plant roots were sampled in third harvest by carefully excavating the plants and breaking the roots into smaller pieces. The samples were first rinsed with diluted water and immersed in a 10% potassium hydroxide solution for 24–72 h. The roots were then washed with dilute water and treated with alkaline hydrogen peroxide for 10–30 min. They were then rinsed again and immersed in a 1% hydrochloric acid solution for 3–5 min before being transferred to a staining solution of lactic acid, glycerol, dilute water, and aniline blue for 24 h. The stained roots were then cut into 1 cm pieces and placed in a petri dish with a grid pattern. The petri dish was examined under a binocular microscope and the intersections between all roots and mycorrhizal roots (identified by their dark blue colour) along the horizontal and vertical grid lines were counted^73^. Root colonization was calculated using the following formula: Root length colonization percentage = (mycorrhizal root length/total root length) × 100.

### Statistical analysis

The process of data analysis was conducted using SAS 9.1 software. The comparison of means was evaluated via LSD (at the significance level of 5%), and the significance comparison of means was obtained by the LS Means test.

### Permission to collect samples

The permission for collection of *Aloe barbadensis* plants was acquired from Agricultural and Natural Resources Ministry of Iran.

### Statement on experimental research and field studies on plants

The *Aloe barbadensis* Mill. plants sampled comply with relevant institutional, national, and international guidelines and domestic legislation of Iran.

## Conclusion

This research was conducted in a region with a hot and dry climate; therefore, superior production stability was seriously pursued for agricultural conservation due to water consumption. The results of the current study showed that *A. barbadensis* has high resistance to drought stress, even in a hot and dry climate. Furthermore, it can be claimed that higher plant growth under each biofertilization level (PGPR, MF and PGPR + MF) occurred because biofertilizers assisted in the increase of plant tolerance and nutrient content. At the same time, antioxidant enzyme activities such as CAT, PRX, and SOD decreased in plants treated with these biofertilizers. In fact, due to reducing the damage of stress and increasing stress tolerance by these biofertilizers in *A.* *barbadensis* plants, the activity of antioxidant enzymes is lower than treatments affected by stress (like non-biofertilizer application treatments). Therefore, the higher nutrient uptake as a result of PGPR and MF inoculation led to better growth and higher drought tolerance. In addition, the best plant conditions in terms of RWC and SFW were achieved with PGPR + MF. It seems that PGPR + MF, among the different biofertilizer treatments, has affected the water status and nutrients in all irrigation conditions, especially under severe stress. Consequently, the combined application of PGPR and MF resulted in an increased SFW or, in other words, an increased productivity of *A. barbadensis.*

## Data Availability

All data generated/analyzed during the study are available with the corresponding author on reasonable request.
